# Hepatitis-B virus reactivation during Immunosuppressive therapy: Clinical observations from a tertiary care center

**DOI:** 10.12669/pjms.42.9.15213

**Published:** 2026-09

**Authors:** Bekir Tunca, Nevin İnce, Şengül Cangür

**Affiliations:** 1 Dr. Bekir Tunca, MD.Associate Professor, Department of Infectious Disease and Clinical Microbiology, Duzce University Faculty of Medicine, Duzce, Turkiye; 2 Dr. Nevin Ince, MD.Associate Professor, Department of Infectious Disease and Clinical Microbiology, Duzce University Faculty of Medicine, Duzce, Turkiye; 3 Prof. Dr. Sengül Cangur, Department of Biostatistics, Duzce University Faculty of Medicine, Duzce, Turkiye

**Keywords:** Anti-HBs titer, Hepatitis B virus, HBV reactivation, Immunosuppressive therapy, Prophylaxis

## Abstract

**Objectives:**

This study evaluated the incidence of hepatitis-B virus reactivation (HBVr) in patients receiving immunosuppressive therapy and examined the association between anti-HBs titers and reactivation risk in routine clinical practice.

**Methodology:**

This retrospective study was conducted at Duzce University Faculty of Medicine Hospital between 2017 and 2020. Adult patients (≥18 years) receiving immunosuppressive therapy and monitored for HBVr were included. A total of 166 patients who were either HBsAg (+) or HBsAg (-) with anti-HBc positivity were analyzed. Demographic and clinical characteristics, type of immunosuppressive therapy, antiviral prophylaxis use, HBV serological markers, HBV DNA levels, and HBVr episodes were obtained from medical records. Patients were categorized into three groups based on their serological status and prophylaxis use: HBsAg (-) / anti-HBc (+) patients not receiving antiviral prophylaxis constituted Group-1 (n=104), HBsAg (-) / anti-HBc (+) patients receiving antiviral prophylaxis constituted Group-2 (n=46), and HBsAg (+) patients receiving antiviral prophylaxis constituted Group-3 (n=16). Statistical analyses were performed using SPSS version 22.0, with p < 0.05 considered statistically significant.

**Results:**

The mean age of the cohort was 54 ± 12.7 years, and 42.1% were male. Most patients were HBsAg (-) / anti-HBc (+) (90.4%), while 9.6% were HBsAg (+). Antiviral prophylaxis was administered to 37.3% of patients. HBVr occurred in 17.3% of HBsAg (-) / anti-HBc (+) patients without prophylaxis and was significantly more frequent in those with anti-HBs titers <100 mIU/mL than in those with titers ≥100 mIU/mL (p = 0.049). Among HBsAg (-) / anti-HBc (+) patients receiving prophylaxis, HBVr was observed in 8.7%. Only one HBVr episode occurred among HBsAg (+) patients receiving prophylaxis.

**Conclusion:**

HBVr risk during immunosuppressive therapy is shaped by HBsAg status, antiviral prophylaxis, and anti-HBs titers. Prophylaxis markedly reduces risk, while anti-HBs titers ≥100 mIU/mL provide additional protection and may guide individualized prophylaxis strategies.

## INTRODUCTION

Hepatitis B virus (HBV) infection remains a major global cause of chronic liver disease; Turkey is classified as a region of moderate endemicity, with an HBsAg seroprevalence of approximately 4% in the adult population.^1^,^2^ Although current antiviral therapies are effective in suppressing hepatitis B virus replication, complete viral clearance is rarely achieved. This is largely due to the persistence of covalently closed circular DNA (cccDNA) within hepatocyte nuclei, which enables the virus to remain in a transcriptionally inactive state and serves as a reservoir for renewed viral replication when host immune control is impaired.^3^,^4^

Although the definition of HBV reactivation (HBVr) varies among studies, it is generally described as the reappearance or increase of HBV DNA levels and/or the reversion to HBsAg positivity (reverse seroconversion) in patients with inactive chronic hepatitis B or resolved HBV infection.^5^–^8^ HBV reactivation may occur in HBsAg (-) / anti-HBc (+) individuals receiving immunosuppressive therapy (IST); therefore, careful assessment of HBV serological status is essential when IST is being considered.^7^,^9^

Current international guidelines uniformly recommend screening all patients scheduled to receive IST for HBV infection using the serological markers HBsAg, anti-HBs, and total anti-HBc; the importance of pre-treatment HBV screening prior to the initiation of biological therapy has been further emphasized in recent real-world studies.^10^,^11^ The risk of HBVr is influenced by the patient’s virological status, host-related factors, and the type, intensity, and duration of IST. Accordingly, guidelines stratify HBVr risk into low, moderate, and high categories based on HBV serology and the immunosuppressive regimen employed.^5^,^8^,^12^ In patients requiring antiviral prophylaxis, initiation of prophylaxis with agents that have a high barrier to resistance is recommended.^7^,^13^

Although HBVr is a well-recognized clinical entity in patients receiving IST, data regarding the impact of anti-HBs levels on the risk of reactivation remain limited. Available studies suggest that anti-HBs positivity and higher antibody titers in patients with resolved HBV infection may be associated with a reduced risk of HBVr. Although the 2025 EASL guidelines recognize anti-HBs titers ≥100 mIU/mL as a factor associated with reduced HBVr risk, no global consensus exists on a specific quantitative threshold to ensure safety during moderate-to-high-risk immunosuppressive therapy.^7^,^14^,^15^

In this retrospective study, HBVr was evaluated in patients receiving IST, with the aim of exploring the association between anti-HBs titers and HBVr, an aspect that has been addressed in a limited number of previous studies.

## METHODOLOGY

This retrospective study included adult patients (≥18 years) receiving IST in various departments of Duzce University Faculty of Medicine Hospital and followed at the Infectious Diseases Outpatient Clinic for HBVr risk between January 2017 and December 2020. A total of 166 patients who were HBsAg **(+)** or HBsAg (-) / anti-HBc (+) with available follow-up data were enrolled.

### Ethical Approval:

This study was conducted after receiving approval from our university’s ethics committee (Approval ID: 2020/01, dated January 20, 2020).

Demographic characteristics, IST types and indications, HBV serological markers, HBV DNA levels, and HBVr status were obtained retrospectively from medical records and the hospital electronic database. Antiviral agents used for prophylaxis were also recorded. Antiviral prophylaxis decisions were made individually based on the patient’s serological status, underlying diagnosis, and the type of immunosuppressive agent used, in the absence of a standardized institutional protocol. Treatment adherence was assessed through clinical evaluation at follow-up visits, as objective measures such as pill counts or pharmacy records were not available.

### Exclusion Criteria:

Patients previously treated for chronic hepatitis B, those with hepatitis C, HIV, or hepatitis D co-infection, those with non-viral liver diseases, individuals with follow-up shorter than six months, patients younger than 18 years, and cases with incomplete data were excluded. Patient enrollment and group allocation are shown in Fig.1.

**Fig.1:**
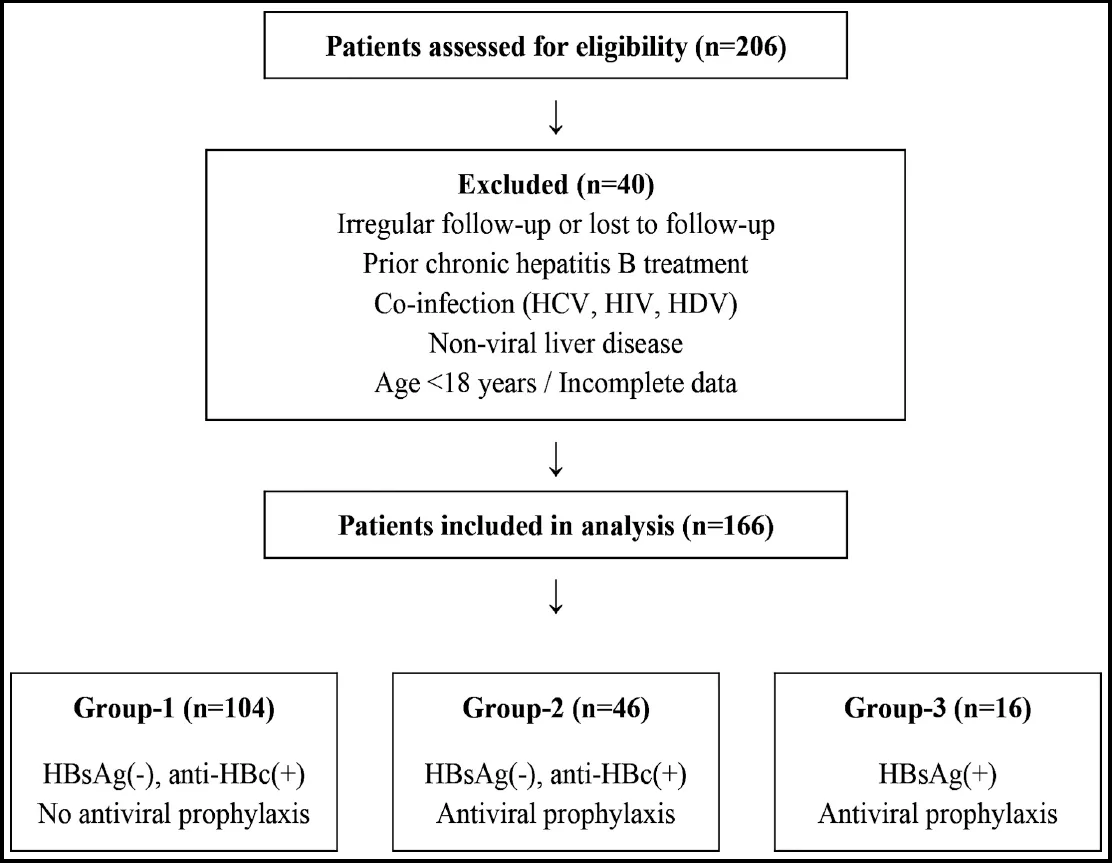
Flow Diagram of Patient Selection and Group Allocation.

### Laboratory tests:

Laboratory monitoring of patients receiving IST and followed for HBVr comprised assessment of HBV serological markers, including HBsAg, HBeAg, anti-HBe, anti-HBs, and total anti-HBc, together with HBV DNA levels and liver biochemical parameters. All serological analyses and HBV DNA testing were conducted at the hospital’s central laboratory using validated commercial assays, with HBV DNA quantification performed by real-time polymerase chain reaction. HBV DNA levels and serological markers were monitored at three-month intervals throughout the duration of immunosuppressive therapy.

### Definition of HBV reactivation:

HBV reactivation in the present study was determined according to predefined, serology-specific criteria. Among HBsAg **(+)** patients, reactivation was considered present when HBV DNA became detectable again or showed an increase of at least tenfold. In contrast, for HBsAg (-) / anti-HBc (+) individuals, HBVr was identified by the occurrence of HBsAg seroconversion and/or the detection of HBV DNA that had previously been undetectable.^4^,^5^ The 100 mIU/mL cut-off value for anti-HBs was selected based on previously published literature suggesting a protective effect at this threshold.

### Statistical analysis:

Study variables were analyzed using appropriate descriptive statistical methods. Measures of central tendency and dispersion were selected according to data characteristics, with continuous variables summarized as either mean values with standard deviations or median values with corresponding ranges. Categorical variables were expressed as counts and proportions. The Shapiro–Wilk test was applied to evaluate distributional assumptions. Statistical analyses were conducted using IBM SPSS Statistics for Windows (version 22.0; IBM Corp., Armonk, NY, USA), and results were considered statistically significant at a two-tailed p value of less than 0.05.

## RESULTS

A total of 166 patients were included in the study. Of these, 70 (42.1%) were male and 96 (57.9%) were female, with a mean age of 54 ± 12.7 years (range: 27–83). Serological evaluation showed that 150 patients (90.4%) were HBsAg(-)/anti-HBc(+), while 16 patients (9.6%) were HBsAg (+) /anti-HBc (+).

Antiviral prophylaxis was administered to 62 patients (37.3%), whereas 104 patients (62.7%) were followed without prophylaxis. The majority of patients were followed in the Physical Medicine and Rehabilitation Clinic (n=152), and the remaining 14 patients were followed in the Dermatology Clinic. All biological agents used in the study were classified as having a moderate risk for HBVr. The demographic characteristics, underlying diagnoses, biological agents, and HBV serological profiles of the patients are summarized in Table-I.

**Table-I:** Demographic, clinical and HBV serological characteristics of patients receiving immunosuppressive therapy (n=166).

Characteristics	n (%)
Age (min-max) (years)	54 ± 12.7 (27-83)
***Gender***	
Male	70 (42.1)
Female	96 (57.8)
***Diagnosis***	
Rheumatoid arthritis	73 (43.9)
Ankylosing spondylitis	62 (37.3)
Psoriatic arthritis	17 (10.2)
Psoriasis	14 (8.4)
HBsAg (+) / anti-HBc (+)	16 (9.6)
HBsAg (-) / anti-HBc (+)	150 (90.4)
***Biological agent class***	
Anti TNF	146 (88)
Anti-IL	13 (7.8)
JAK inhibitor	7 (4.2)

Anti-TNF: tumour necrosis factor inhibitors,

Anti-IL: interleukin inhibitors, JAK: Janus kinase,

HBV: Hepatitis B virus;

HBsAg: Hepatitis B surface antigen;

anti-HBc: Hepatitis B core antibody;

anti-HBs: Hepatitis B surface antibody.

Based on serological status and antiviral prophylaxis use, patients were classified into three groups:

- HBsAg (-) / anti-HBc (+) patients without antiviral prophylaxis (Group-1, n=104)
- HBsAg (-) / anti-HBc (+) patients receiving antiviral prophylaxis (Group-2, n=46)
- HBsAg (+) patients receiving antiviral prophylaxis (Group-3, n=16)

Among the 104 HBsAg (-) / anti-HBc (+) patients who did not receive antiviral prophylaxis, HBVr was detected in 18 patients (17.3%). Patients with and without HBVr were compared according to sex, age (<65 vs. ≥65 years), indication for initiation of IST, type of biological agent, and anti-HBs titer (<100 vs. ≥100 mIU/mL). There were no significant differences between the groups in terms of sex, age group, underlying diagnosis, or the biological agent used (p values >0.05 for all comparisons).

No cases of HBsAg reverse seroconversion or biochemical hepatitis were observed; reactivation was limited to the virological level and defined solely by the detection of HBV DNA (>10 IU/mL). The rate of HBVr was significantly higher in patients with anti-HBs titers <100 mIU/mL compared with those with titers ≥100 mIU/mL (p=0.049). This association is summarized in Table-II.

**Table-II:** Clinical characteristics associated with HBV reactivation in HBsAg (-) / anti-HBc (+) patients not receiving antiviral prophylaxis (Group-1, n=104)

Characteristics		HBVr						p value
		Present (n:18)		Absent (n:86)		Total (n:104)		
		n	%	n	%	n	%	
Gender	Female	9	19.6	37	80.4	46	100	0.588*
	Male	9	15.5	49	84.5	58	100	
Age	<65	12	16.2	62	83.8	74	100	0.644*
	≥65	6	20.0	24	80.0	30	100	
Diagnosis	Rheumatoid arthritis	7	14.6	41	85.4	48	100	0.461^#^
	Ankylosing spondylitis	8	21.1	30	78.9	38	100	
	Psoriasis	2	33.3	4	66.7	6	100	
	Psoriatic arthritis	1	8.3	11	91.7	12	100	
Biological agent class	Anti-TNF	15	16.3	77	83.7	92	100	0.545^#^
	Anti-IL	2	25	6	75	8	100	
	JAK inhibitor	1	25	3	75	4	100	
Anti-HBs (mIU/ml)	≥100	7	11.3	55	88.7	62	100	0.049*
	<100	11	26.2	31	73.8	42	100	
	Total	18	17.3	86	82.7	104	100	

*: Pearson’s chi-square test, #: Fisher-Freeman-Halton test HBVr, hepatitis B virus reactivation;

Anti-TNF, tumour necrosis factor inhibitors; Anti-IL, interleukin inhibitors; JAK, Janus kinase.

To further evaluate the independent association between anti-HBs titer and HBVr, a multivariate logistic regression analysis was performed including sex, age (≥65 years), underlying diagnosis, biological agent class, and anti-HBs titer (<100 mIU/mL) as covariates. Anti-HBs titer <100 mIU/mL was identified as a potential independent predictor of HBVr (OR: 3.163, 95% CI: 1.064–9.397, p=0.038), while sex, age, diagnosis, and biological agent class were not independently associated with reactivation risk. The results are presented in Table-III.

**Table-III:** Multivariate logistic regression analysis of factors associated with HBV reactivation in HBsAg (-) / anti-HBc (+) patients without antiviral prophylaxis (Group-1, n=104).

Variable	OR	95% CI	p
Sex (female vs male)	0.923	0.294–2.902	0.892
Age ≥65 years	0.809	0.231–2.833	0.740
Diagnosis	—	—	0.495
Biological agent class	—	—	0.677
Anti-HBs <100 mIU/mL	3.163	1.064–9.397	0.038*

Diagnosis (rheumatoid arthritis, ankylosing spondylitis, psoriasis, psoriatic arthritis) and biological agent class (anti-TNF, anti-IL, JAK inhibitor) were included as categorical covariates; individual subgroup OR values are not shown due to limited subgroup sizes. OR, odds ratio; CI, confidence interval; Anti-HBs, hepatitis B surface antibody; Anti-TNF, tumour necrosis factor inhibitors; Anti-IL: interleukin inhibitors; JAK, Janus kinase.

HBVr was detected in 4 out of 46 patients in Group-2 (8.7%). In these patients, reactivation was limited to the virological level, confined to HBV DNA positivity; no HBsAg seroconversion or biochemical hepatitis findings were observed. Two of the patients who developed HBVr were receiving TDF prophylaxis, and two were receiving ETV prophylaxis. The HBV DNA levels of the four patients who developed virological reactivation despite prophylaxis were 2,860, 120, 1,460, and 140 IU/mL, respectively, indicating low-level reactivation. Reactivation was attributed to irregular use of prophylaxis, and HBV DNA tested negative in all four patients at the three-month follow-up. When patients who developed HBVr and those who did not were compared in terms of gender, age, underlying disease diagnosis, antiviral agent used, and anti-HBs titer ≥100 mIU/mL, no statistically significant differences were found between the variables. Analysis by type of biological agent could not be performed due to the limited number of patients who developed HBVr; the relevant comparisons are presented in Table- IV.

**Table-IV:** Clinical characteristics associated with HBV reactivation in HBsAg (-) / anti-HBc (+) patients receiving antiviral prophylaxis (Group-2, n=46).

Characteristics		HBVr						P value
		Present (n:4)		Absent (n:42)		Total (n:46)		
		n	%	n	%	n	%	
Gender	Female	1	5.6	17	94.4	18	100	0.999^&^
	Male	3	10.7	25	89.3	28	100	
Age	<65	3	9.7	28	90.3	31	100	0.999^&^
	≥65	1	6.7	14	93.3	15	100	
Diagnosis	Rheumatoid arthritis	2	10	18	90	20	100	0.842^#^
	Ankylosing spondylitis	1	5.9	16	94.1	17	100	
	Psoriasis	1	16.7	5	83.3	6	100	
	Psoriatic arthritis	0	0	3	100	3	100	
Biological agent class	Anti-TNF	3	7.1	39	92.9	42	100	0.314^#^
	Anti-IL	1	33.3	2	66.7	3	100	
	JAK inhibitor	0	0	1	100	1	100	
Antiviral prophylaxis	TDF	2	13.3	13	86.6	15	100	0.699^#^
	TAF	0	0	3	100	3	100	
	ETV	2	7.1	26	92.8	28	100	
Anti-HBs (mIU/ml)	≥100	1	8.3	11	91.7	12	100	0.999^&^
	<100	3	8.8	31	91.2	34	100	
	Total	4	8.7	42	91.3	46	100	

&: Fisher’s Exact Test, #: Fisher-Freeman-Halton Test, HBVr, hepatitis B virus reactivation;

TDF, tenofovir disoproxil fumarate; TAF, tenofovir alafenamide; ETV, entecavir;

Anti-TNF, tumour necrosis factor inhibitors; Anti-IL, interleukin inhibitors; JAK, Janus kinase.

Of the 16 patients in Group-3, TDF was used in eight, TAF in one, ETV in two, and lamivudine (LAM) in five as prophylaxis. HBVr was detected in one patient receiving LAM. Due to the limited number of patients who developed HBVr, statistical analysis could not be performed with respect to age, underlying disease, biological agent used, and type of antiviral prophylaxis; the relevant findings are presented in Table-V.

**Table-V:** Clinical characteristics associated with HBV reactivation in HBsAg (+) patients receiving antiviral prophylaxis (Group-3, n=16)

Characteristics		HBVr						P value
		Present (n:1)		Absent (n:15)		Total (n:16)		
		n	%	n	%	n	%	
Gender	Female	1	16.7	5	83.3	6	100	0.375^&^
	Male	0	0	10	100	10	100	
Age	<65	1	6.3	15	93.8	16	100	-
	≥65	0	0	0	0	0	0	
Diagnosis	Rheumatoid arthritis	0	0	5	100	5	100	-
	Ankylosing spondylitis	1	14.3	6	85.7	7	100	
	Psoriasis	0	0	2	100	2	100	
	Psoriatic arthritis	0	0	2	100	2	100	
Biological agent class	Anti-TNF	1	8.3	11	91.7	12	100	-
	Anti-IL	0	0	2	100	2	100	
	JAK inhibitor	0	0	2	100	2	100	
Antiviral prophylaxis	TDF	0	0	8	100	8	100	-
	TAF	0	0	1	100	1	100	
	ETV	0	0	2	100	2	100	
	LAM	1	20	4	80	5	100	
	Total	1	6.3	15	93.8	16	100	

&: Fisher Exact test, HBVr, hepatitis B virus reactivation; TDF, tenofovir disoproxil fumarate;

TAF, tenofovir alafenamide; ETV, entecavir; LAM, lamivudine;

Anti-TNF, tumour necrosis factor inhibitors; Anti-IL, interleukin inhibitors; JAK, Janus kinase.

## DISCUSSION

Among patients with previous exposure to hepatitis B virus who undergo IST without antiviral prophylaxis, published data indicate a considerable variability in HBVr rates, with reported frequencies ranging between 3.5% and 27.3%.^16^ In the present study, patient grouping was based on HBsAg status in conjunction with the use of antiviral prophylaxis. Individuals who were HBsAg (-) / anti-HBc (+) were divided into two subgroups according to prophylaxis status, namely those without antiviral prophylaxis (Group-1) and those receiving prophylaxis (Group-2). Patients who were HBsAg (+) and treated with oral antiviral prophylaxis were analyzed separately as Group-3. In the present analysis, HBVr occurred in 17.3% of patients in Group-1 and in 8.7% of those in Group-2. These findings are consistent with previously published data.^16^ Findings from a meta-analysis including 468 patients with resolved hepatitis B infection showed that HBVr was observed in eight cases, corresponding to a rate of 1.7%.^17^ According to data published by Perez-Alvarez et al., HBVr was observed in 5.3% of individuals with resolved HBV infection who were receiving anti-TNF therapy, a rate that was lower than that found in our study population.^18^ Consistent with our findings, an HBVr rate of 17.2% has also been reported by Solay et al.^19^

Our study included a total of 46 patients in Group-2 who were HBsAg (-)/ anti-HBc (+) and receiving antiviral prophylaxis. Within this cohort, antiviral prophylaxis consisted of TDF in 15 patients, ETV in 28 patients, and TAF in three patients. HBV reactivation was identified in only four individuals (8.7%) in this group, indicating a lower likelihood of reactivation among patients receiving prophylaxis. By comparison, HBVr occurred in 17.3% of patients in Group-1 who were not given antiviral prophylaxis, further suggesting a protective role of prophylactic antiviral therapy. In the four patients who developed HBVr despite prophylaxis, reactivation was limited to virological findings, with no evidence of HBsAg seroreversion or biochemical hepatitis. In this context, the lower incidence of HBVr observed in Group-2 (8.7%) compared with Group-1 supports both the effectiveness of the antiviral agents used and the role of antiviral prophylaxis as a preventive strategy against HBVr. HBVr occurred in two patients receiving TDF and two receiving ETV, and non-adherence to therapy was considered a possible contributing factor. Consistent with these findings, a study from our country reported HBVr in only one of 199 patients receiving antiviral prophylaxis, which was attributed to treatment discontinuation.^20^

Accumulating evidence suggests that both the presence and magnitude of anti-HBs antibodies may exert a protective effect against HBVr. Supporting this concept, a meta-analysis involving 1,672 HBsAg (-) / anti-HBc (+) patients treated for hematological malignancies demonstrated a markedly higher reactivation rate among anti-HBs (-) individuals (14%) compared with those who were anti-HBs **(+)** (5%).^14^ In a meta-analysis, the overall risk of HBVr among HBsAg (-) / anti-HBc (+) patients receiving rituximab therapy was reported to be 12.6% (95% CI: 8.3–18.8%); however, the risk of reactivation varied significantly according to anti-HBs status and titer, with the highest risk observed in anti-HBs (-) patients (27.3%), decreasing to 13.8% in those with anti-HBs levels <100 IU/mL, and reaching a markedly low rate of 3.5% in patients with anti-HBs levels ≥100 IU/mL.^16^ In our study, when patients with anti-HBs levels ≥100 mIU/mL and <100 mIU/mL were compared, it was found that HBVr developed significantly more in patients with low anti-HBs titers (<100 mIU/mL) (p<0.05); this finding was consistent with studies reporting that high anti-HBs titers may be protective against HBVr. Although the difference in HBVr rates based on anti-HBs titers reached borderline statistical significance in univariate analysis (p=0.049), multivariate logistic regression analysis further supported this association, identifying an anti-HBs titer <100 mIU/mL as a potential independent predictor of HBVr (OR: 3.163, 95% CI: 1.064–9.397, p=0.038). To the best of our knowledge, this is one of the few studies to evaluate a specific anti-HBs titer threshold as an independent predictor of HBVr using multivariate analysis in a real-world clinical setting; moreover, data from intermediate-endemicity regions such as Turkey remain underrepresented in the literature. These findings should nonetheless be interpreted with caution given the borderline significance and limited sample size, and heterogeneity in immunosuppressive drug doses and treatment durations may have further influenced reactivation risk. Future prospective, multicentre studies with larger patient cohorts are needed to validate the 100 mIU/mL threshold and inform evidence-based clinical guidelines.

International guidelines recommend the use of TDF, TAF or ETV for antiviral prophylaxis prior to IST in HBsAg **(+)** patients.^10^ In contrast, long-term use of lamivudine is associated with high rates of antiviral resistance; reported studies have shown resistance rates of 14-30% in the first year, reaching 70% in the fifth year.^21^ In a study by Huang et al., HBVr was observed in 18 of 60 HBsAg **(+)** individuals receiving R-CHOP chemotherapy with lamivudine prophylaxis (30%), whereas reactivation was detected in only four of 61 individuals (6.6%) receiving entecavir prophylaxis.^22^ In our study, antiviral prophylaxis was initiated in all 16 HBsAg **(+)** patients (Group-3), and antiviral treatment was continued with TDF after HBVr was detected in only one patient receiving lamivudine prophylaxis.

### Limitations:

This study has some limitations. The study population is limited to patients followed up in dermatology and physical medicine and rehabilitation clinics who are in the intermediate risk group for HBVr according to current guidelines; patients with haematological malignancies and those receiving more intensive immunosuppression were not evaluated. Furthermore, the retrospective and single-centre design, along with the limited sample size, restricts the generalisability of the results. Other limitations of the study include the inability to assess treatment adherence using objective methods, the lack of HBV resistance testing, and the definition of HBVr based solely on virological markers without evidence of severe clinical or biochemical hepatitis. Furthermore, the borderline statistical significance observed at the anti-HBs titer cutoff of <100 mIU/mL suggests that larger patient cohorts may be needed to demonstrate a more pronounced difference. Therefore, prospective, multicentre studies involving larger patient groups are needed to validate our findings.

## CONCLUSIONS

Our study demonstrates that the risk of HBVr in patients receiving IST depends not only on HBsAg status but also on the use of antiviral prophylaxis and the quantitative level of anti-HBs titers. It was found that antiviral prophylaxis significantly reduced the risk of HBVr in HBsAg (-) / anti-HBc (+) patients, while anti-HBs levels ≥100 mIU/mL provided an additional protective effect. These findings suggest that integrating anti-HBs titers into clinical decision-making processes in patients at moderate risk may contribute to optimising antiviral prophylaxis strategies.

### Authors Contribution:

**BT and NI** did data collection, literature search, revision of the final draft.

**SC** did statistical analysis. **NI** responsible for the accuracy of the study.

All authors have read and approved the final manuscript.
